# Intestinal Mycobacterium avium Complex Infection in a Kidney Transplant Patient

**DOI:** 10.7759/cureus.28007

**Published:** 2022-08-14

**Authors:** Corbin Walters, Dipa Puwar, Chirag Patel, Daniel Eshaghian, Vasudevan Unnithan Raghuraman

**Affiliations:** 1 Internal Medicine, Oklahoma State University Medical Center, Tulsa, USA; 2 Gastroenterology, Saint Francis Medical Center, Tulsa, USA

**Keywords:** duodenal biopsy, infectious disease and gastroenterology, mycobacterium avium-complex, kidney transplantation recipients, gastroenterology and endoscopy

## Abstract

Opportunistic infections are the result of infection by bacteria, viral, and fungal sources potentially leading to severe disease and death. These infections are a cause of significant morbidity and mortality among individuals with profound immunosuppression, namely human immunodeficiency virus (HIV), and organ transplant recipients on medications used to prevent organ rejection. *Mycobacterium avium *complex (MAC) is one of the most prevalent pathogens worldwide as it is found ubiquitously in water, food, and soil and is commonly a source of disseminated disease among the immunocompromised. However, cases of kidney transplantation remain exceedingly rare with an estimated incidence of 0.16% and 0.55%. We present the case of a 68-year-old female with a history of a kidney transplant, currently on immunosuppressant therapy, who was found to have localized MAC infection after undergoing endoscopic evaluation for symptoms of generalized weakness and unintentional weight loss secondary to anemia.

## Introduction

Mycobacterium avium complex (MAC) is one of the most prevalent pathogens worldwide. Due to the sheer number of mycobacterium species causing disease, the exact number of infections remains unknown; however, it is estimated in the United States that the annual incidence and prevalence are 4.73 and 11.70 cases per 100,000 persons, respectively [1]. A recent study reported a progressively rising number of MAC infections among United States citizens [1], with a similar trend observed internationally [2]. A likely explanation for the increased case volume is the growing number of immunocompromised individuals. As the most common type of nontuberculous mycobacteria, MAC infection occurs as a result of bacterial invasion through mucosal surfaces. Sources of MAC are located ubiquitously in water, food, and soil, with a predilection for severe disease seen in the immunocompromised, including those with lung disease, autoimmune disorders, and human immunodeficiency virus (HIV), and transplant recipients among other causes of immune system suppression [3].

MAC generally infects the lungs in individuals with and without underlying pulmonary disease leading to increased shortness of breath and nonproductive cough, but may less frequently cause disseminated disease resulting in a variety of symptoms based on the organs affected. Other nonspecific symptoms such as fever, night sweats, weight loss, and gastrointestinal problems are also commonly observed [3]. Diagnosis of MAC infection relies upon microscopic evidence consisting of MAC-positive blood cultures or samples cultured from sputum or bronchoalveolar washings in the case of primary pulmonary disease and fluid or tissue specimens from sites of suspected involvement in disseminated disease [4]. Cultures may take up to six weeks to reveal bacterial growth. Notably, the symptomatology and diagnostic approach for MAC infection is similar to that of mycobacterium tuberculosis, which similarly may stain acid-fast positive, but can be differentiated by the presence of noncaseating granulomas upon histological examination. Radiographic evidence may reveal nodular or cavitations on chest radiography and lesions of seeded organs; however, these findings are relatively nonspecific. Guideline-directed treatment consists of a macrolide, ethambutol, and a rifamycin for 6-12 months and addresses underlying immunodeficiencies [4].

Disseminated MAC occurs through the seeding of organs and tissues causing multifactorial disease. Rarely, MAC can infect the gastrointestinal system resulting in significant morbidity and occasionally death [5,6]. In this case report, we present the case of disseminated enteritis and colitis secondary to MAC infection in a woman with a history of renal transplant taking chronic immunosuppressive therapy.

## Case presentation

We present the case of a 68-year-old female with a history of kidney transplant complicated by chronic transplant glomerulonephropathy, currently on immunosuppressant therapy of cyclosporine, prednisone, and mycophenolate mofetil, that presented to the emergency department upon request of her primary care provider for abnormal laboratory findings. Her laboratory findings were significant for a hemoglobin of 7.6 g/dL (normal: 11.6-15 g/dL), potassium of 5.1 mmol/L (normal: 3.5-4.5 mmol/L), calcium of 13 mg/dL (normal: 8.6-10.3 mg/dL), BUN of 51 mmol/L (normal: 2.1-8.5 mmol/L), and creatinine of 3.33 mg/dL (normal: 0.6-1.1 mg/dL), notably worse from her baseline creatinine of 2.6 mg/dL. The patient reported symptoms of generalized weakness and unintentional weight loss of nearly 14 kg over a period of approximately three months. A physical exam revealed prominent pallor with diffuse muscle and temporal wasting. She denied any respiratory complaints including shortness of breath, cough, or hemoptysis.

**Figure 1:**
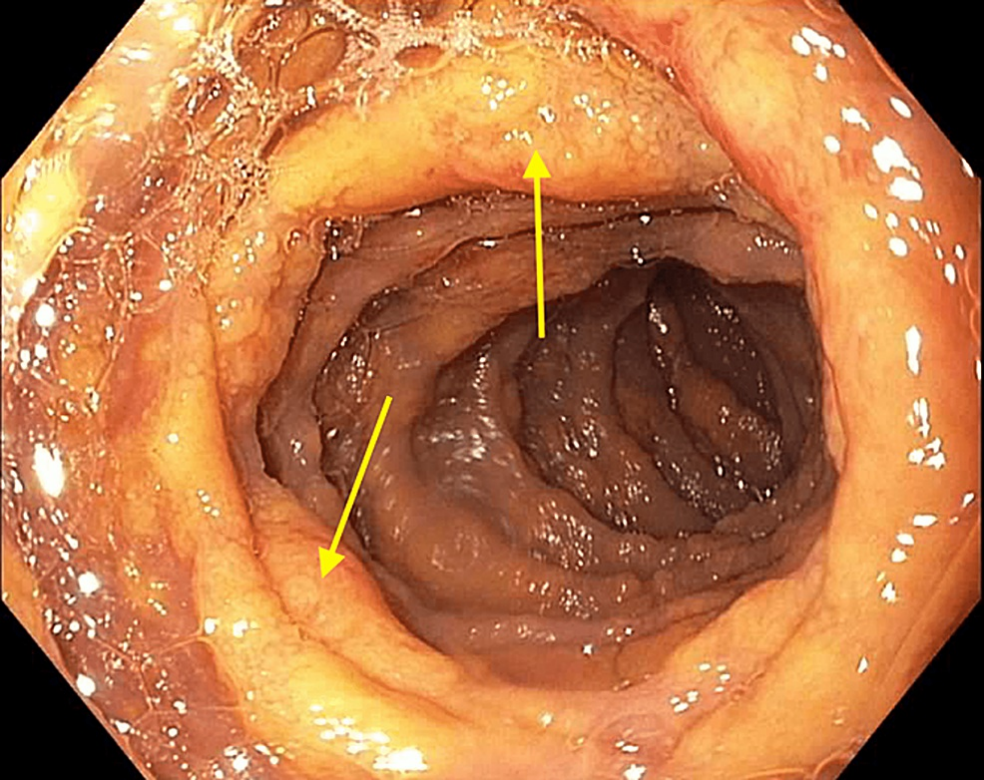
Second portion of the duodenum showing granular appearance with plaque formation (arrows)

During her hospitalization, the patient experienced worsening weakness. The repeat hemoglobin level was 6.1 g/dL, so she was transfused 250 mL of packed red blood cells. Computed tomography (CT) of the abdomen and pelvis revealed prominent wall thickening in the cecum and ascending colon with diffuse abdominal lymphadenopathy. The Gastroenterology service was consulted for symptomatic anemia and the findings of the CT. The patient was scheduled for esophagogastroduodenoscopy (EGD) and colonoscopy. A previous colonoscopy in 2011 showed non-bleeding internal hemorrhoids, sigmoid diverticulosis, and a single non-bleeding colonic angiodysplastic lesion. She denied undergoing a previous EGD. On EGD, she was found to have grade C esophagitis, grade 1 esophageal varices, a 5-cm hiatal hernia, and diffuse gastritis that was negative for Helicobacter pylori-negative based on CLOtest biopsy. Patchy, granular mucosa with yellow plaque deposition was observed throughout the entirety of the duodenum (Figure 1).

**Figure 2:**
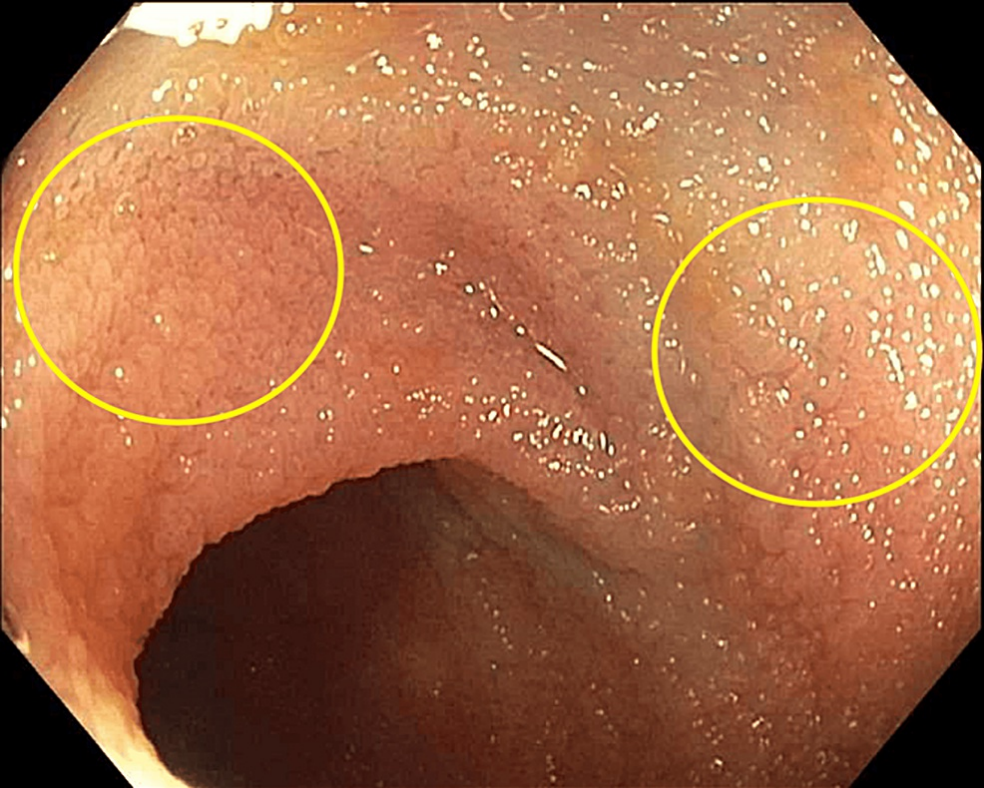
Terminal ileum showing granular appearance with plaque formation (circles)

Multiple biopsies of these lesions were taken for further evaluation. On colonoscopy, the patient was found to have multiple polyps in the cecum, ascending colon, transverse colon, descending colon, and sigmoid colon that were removed by a snare. A similar plaque deposition was seen throughout the large intestine and terminal ileum (Figure 2).

**Figure 3:**
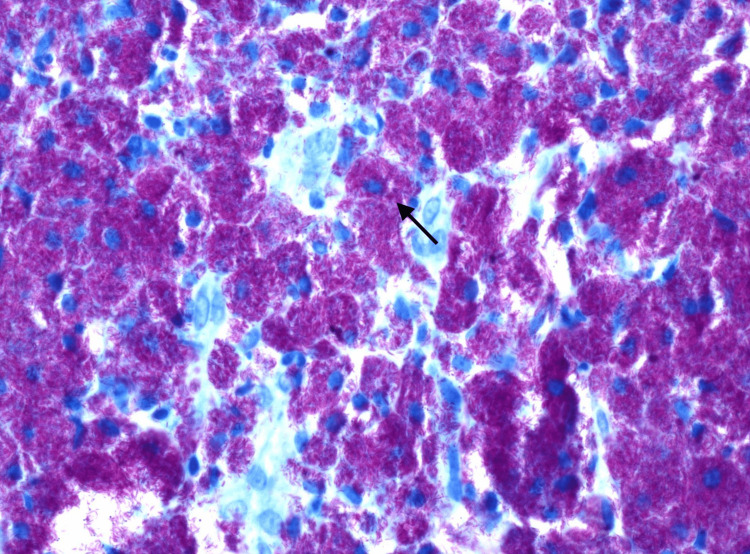
Rod-shaped bacilli with Kinyoun AFB stain (arrow) shown densely packed within histiocytes of the colon, consistent with Mycobacterium avium complex (400x magnification)

These lesions were also biopsied for further evaluation. Histological examination (Figure 3) and culture of these biopsies were consistent with *M. avium* complex infection. Based on antibiotic susceptibility testing collected from the biopsies, the patient was started on azithromycin, ethambutol, and rifabutin, and the dosages of her immunosuppressive regimen were decreased to prevent toxicity. Despite the initiation of therapy, the patient continued to report significant debility secondary to generalized weakness and was discharged to a rehabilitation facility for further recovery.

## Discussion

It is estimated that between 20% and 40% of individuals with HIV with CD4 T lymphocyte (CD4) cell counts <50 cells/mm^3^ will develop disseminated MAC infection [7]. However, similar estimates among individuals with other forms of profound immunosuppression, such as solid organ transplant recipients, are not well documented. The use of prophylaxis against MAC remains controversial and is not currently recommended among HIV patients [7] and no formal recommendations exist among transplant recipients. Treatment considerations do not differ between the mentioned populations, consisting of a minimum of 12 months of a macrolide, ethambutol, and rifamycin [4]. Caution is warranted in order to minimize medication interactions between antimycobacterial agents and immunosuppressants, such as calcineurin inhibitors and sirolimus. These interactions can result in increased concentrations of immunosuppressants; therefore, drug levels should be closely monitored with close consultation with infectious disease and transplant specialists. In this case, the patient was prescribed azithromycin, ethambutol, and rifabutin and her immunosuppressive regimen was correspondingly decreased. Following guideline recommendations [4], these medications were selected based on her susceptibility testing for a minimum of 12 months. The treatment is considered successful with completion of the antimycobacterial regimen, total resolution of her symptoms, and correction of laboratory abnormalities due to MAC infection, including hypercalcemia and worsening anemia. Notably, some patients may require an extended duration of therapy or different regimens based on susceptibility testing or specialist recommendation.

The patient in this case presented with non-specific symptoms and profound anemia prompting gastroenterology consultation to rule out a gastrointestinal source of blood loss. Upon endoscopic exam, the patient demonstrated diffuse patchy infiltrates throughout much of the small and large intestines. Biopsy of these infiltrates revealed disseminated MAC infection of the gastrointestinal tract. This initial presentation of MAC infection in the absence of pulmonary symptoms is exceedingly rare. It is proposed that these organisms are phagocytosed by the macrophages of the lamina propria of the small intestines, spreading throughout much of the submucosal tissue before being transported systemically by the lymphatic system and the bloodstream [5]. Hematogenous dissemination of MAC to the bone marrow and spleen is common, resulting in anemia from hematopoietic suppression and hypercalcemia secondary to granulomatous disease, respectively [5]. Both of these lab abnormalities were seen in this case and are expected to resolve following successful treatment.

Opportunistic infections of the gastrointestinal tract are exceedingly common. Susceptibility to these organisms is believed to be the result of disruption to the healthy, commensal bacteria of the gastrointestinal tract in the setting of impaired host immune function, which favors the overgrowth of pathogenic organisms [7]. This imbalance may present as a spectrum of findings, including oral and esophageal lesions associated with Candidiasis, diffuse gastrointestinal tract lesions seen with viral infections, hepatobiliary lesions, pancreatitis, and anorectal lesions [8,9]. Among individuals with HIV, these conditions present along a continuum corresponding to the person’s CD4 count [10]. Contrastingly, an easily recognized continuum of gastrointestinal opportunistic infections is not observed among organ transplant recipients. Therefore, thoughtful consideration is warranted for the treatment of gastrointestinal opportunistic infections in these patients, as initial presenting symptoms may be vague and non-specific as was the instance with the patient presented in this case [11]. Multimodal diagnostic techniques, such as EGD, capsule endoscopy, and colonoscopy with biopsy may aid in differentiating common opportunistic infections of the gastrointestinal tract [12,13].

## Conclusions

In conclusion, we present the diagnosis and management of a patient presenting with disseminated MAC infection detected by endoscopic biopsy. It is important to consider MAC among other opportunistic infections of the gastrointestinal tract, such as cytomegalovirus, Ebstein-Barr virus, and herpesvirus in immunosuppressed patients. Treatment considerations are similar to infected immunocompetent persons, but the immunosuppressed will require an extended duration of therapy and close monitoring of medication interactions.
